# Prevalence and prediction of dropout during depression treatment in routine outpatient care: an observational study

**DOI:** 10.1007/s00406-022-01499-1

**Published:** 2022-10-17

**Authors:** D. A. van Dijk, M. L. Deen, Th. M. van den Boogaard, H. G. Ruhé, J. Spijker, F. P. M. L. Peeters

**Affiliations:** 1 Department of Clinical Psychological Science, Faculty of Psychology and Neuroscience, Maastricht University, Maastricht, The Netherlands; 2 Department of Mood Disorders, PsyQ Haaglanden, The Hague, The Netherlands; 3 Parnassia Psychiatric Institute, The Hague, The Netherlands; 4 Institute of Psychology, Leiden University, Leiden, The Netherlands; 5 Department of Psychiatry, Radboudumc, Nijmegen, The Netherlands; 6 Donders Institute for Brain and Behaviour, Radboud University, Nijmegen, The Netherlands; 7 Behavioural Science Institute, Radboud University, Nijmegen, The Netherlands; 8 Pro Persona Mental Healthcare, Nijmegen, The Netherlands; 9 Parnassia Psychiatric Institute, Monsterseweg 93, 2553 RJ The Hague, The Netherlands

**Keywords:** Depressive disorder, major, Dropout, Outpatients, Cohort studies, Treatment outcome

## Abstract

Efficacious treatments are available for major depressive disorder (MDD), but treatment dropout is common and decreases their effectiveness. However, knowledge about prevalence of treatment dropout and its risk factors in routine care is limited. The objective of this study was to determine the prevalence of and risk factors for dropout in a large outpatient sample. In this retrospective cohort analysis, routinely collected data from 2235 outpatients with MDD who had a diagnostic work-up between 2014 and 2016 were examined. Dropout was defined as treatment termination without achieving remission before the fourth session within six months after its start. Total and item scores on the Dutch Measure for Quantification of Treatment Resistance in Depression (DM-TRD) at baseline, and demographic variables were analyzed for their association with dropout using logistic regression and elastic net analyses. Data of 987 subjects who started routine outpatient depression treatment were included in the analyses of which 143 (14.5%) dropped out. Higher DM-TRD-scores were predictive for lower dropout odds [OR = 0.78, 95% CI = (0.70–0.86), *p* < 0.001]. The elastic net analysis revealed several clinical variables predictive for dropout. Higher SES, higher depression severity, comorbid personality pathology and a comorbid anxiety disorder were significantly associated with less dropout in the sample. In this observational study, treatment dropout was relatively low. The DM-TRD, an easy-to-use clinical instrument, revealed several variables associated with less dropout. When applied in daily practice and combined with demographical information, this instrument may help to reduce dropout and increase treatment effectiveness.

## Introduction

Despite the existence of efficacious treatments for major depressive disorder (MDD), premature treatment discontinuation (dropout) by patients restricts their effectiveness [1]. Treatment dropout should be distinguished from treatment refusal, where patients fail to start the intervention that was offered which is another type of problem with different rates and moderators [2]. Dropout is not only associated with worse short- and long-term clinical outcomes for patients [1] but also has a negative impact on their social functioning and care providers [3, 4]; and impedes effective use of scarce resources in mental health [5]. Regardless of slightly different definitions, most studies report that dropout occurs primarily early in treatment [6–8]. Early identification of expected treatment dropout might reduce occurrence thereof and increase treatment effectiveness.

Dropout rates for psychotherapy in depression treatment are estimated to be around 20% (range 0–74%) [6, 9]. Rates for pharmacotherapy may vary from around 30% in clinical trials to as high as 60% in naturalistic settings [10–12]. Many efforts have been made to identify factors that are associated with dropout of treatment, with inconsistent findings. The demographic characteristics race (non-Caucasian), less education and lower socio-economic status (SES) are regularly associated with more dropout [5, 6, 9, 10, 13–15]. The results of studies examining the relationship between other demographic characteristics such as age, marital status and gender are less convincing. Male gender [16–19], younger age [9, 13, 16–20], and not being in a relationship [15, 17] have been associated with dropout. However, results are inconclusive according to others [6, 14, 21]. These inconsistencies also apply to the relationship between clinical features and the occurrence of dropout. The impact of higher baseline severity on the occurrence of dropout for example has not been consistently established, ranging from protective [5, 8, 22–24] or no clear impact [13] to risk enhancing [19, 21, 25–27]. Recurrent depression is found to be associated with lower dropout rates [28], whereas chronic depression may not affect the occurrence of dropout [21]. Co-morbidity such as personality disorders and eating disorders [6, 9, 29] are considered to be risk enhancing by some, but other studies describe no or even a protective effect of personality pathology [13, 21]. Inconsistencies are also apparent in studies on the influence of comorbid anxiety in MDD treatment outcomes including dropout [13, 30, 31]. Treatment-related variables like higher patient (pre-) treatment expectancies, patients receiving their preferred treatment and a good therapeutic alliance as well as a more experienced therapist are associated with less dropout. [9, 32–35].

Studies directed at establishing treatment effectiveness reported higher dropout rates (26%) in comparison to randomized studies designed for treatment efficacy (17%) [9]. In fact, the majority of empirical knowledge on dropout may not be useful for daily clinical practice, being derived from randomized controlled trials (RCT’s). Hans & Hiller (2013) conducted a meta-analysis of non-randomized effectiveness studies of CBT for depression and reported a dropout rate of 24.6% with a wide range between studies (0–68%). Therefore, dropout from treatment in naturalistic treatment settings should be examined separately from investigations in traditional RCT oriented research contexts such as university affiliated settings [36, 37].

Identification of risk factors for dropout in routine care using a feasible measure will be helpful to prevent its occurrence and increase effectiveness of depression treatment. In the current retrospective study, we aimed to quantify risk factors for treatment dropout in a large naturalistic sample of outpatients with depression receiving treatment in a routine treatment facility in secondary care. We hypothesized that dropout rate would exceed 20% as typically found for effectiveness trials. Next, we hypothesized that known risk factors for treatment resistance (i.e., severity and duration of the depressed episode) would be associated with more dropout. Moreover, in line with mainly meta-analytic evidence, we expected male gender, younger age, lower social economic state, and the presence of a comorbid anxiety or personality disorder to be positively associated with dropout.

## Methods

### Design and participants

Data were derived from PsyQ, a nationwide outpatient secondary mental healthcare provider in the Netherlands. In these healthcare settings treatment (psychotherapy, pharmacotherapy or both) is provided according to the Dutch Multidisciplinary Guideline for Depression [38]. Seven locations^1^ participated in this study. We only used anonymized data retrospectively extracted from the Electronic Patient Record (EPR). The Medical Research Ethics Committee of the Leiden University Medical Centre waived formal informed consent for the use of this data since subjects were not exposed to any experimental intervention [39]. Data from individuals who objected against the use of their information for scientific purposes, before or during treatment, were not included.

We included patients 18 years or older and meeting DSM-IV-TR [40] criteria for MDD or dysthymic disorder as their primary diagnosis according to the Mini-International Neuropsychiatric Interview (M.I.N.I.) [41]. Data concerning gender, age and marital state were extracted from the EPR. The socio-economic status (SES) was determined by the ‟Statusscores 2017” from the Netherlands Institute of Social Research, which represent the SES score of all Dutch zip-code areas compared to the reference score of 0 within a range from −7.78 to + 2.82. We included data of subjects who had a comprehensive diagnostic work-up, i.e., an interview with a clinician in which the diagnosis has been made and the DM-TRD (see below) was fully completed, between June 2014 and June 2016. Subjects that had a baseline (± 30 days from diagnostic work-up) score on the Quick Inventory of Depressive Symptomatology—Self Report (QIDS-SR) of less than 11 (moderate depression) were excluded from our analyses. This threshold of at least 11 was chosen to minimize the chance that the occurrence of dropout would be the result of a lack of treatment motivation due to limited severity of the depression. We excluded patients with bipolar disorders, psychotic disorders or substance dependence (except for nicotine).

### Measures

#### Quick inventory of depressive symptomatology—self-report (QIDS-SR)

The QIDS-SR is a 16-item self-report depression symptom severity measure derived from the 30-item Inventory of Depressive Symptoms (IDS) [42]. QIDS-SR scores vary between 0 and 27, where ≤ 5 is defined as ‘no depression’ (or ‘remission’ in formerly depressed subjects) and scores ≥ 21 are defined as ‘very severe depression’, where intermediate scores indicate the categories ‘mild’, ‘moderate’ and ‘severe’. The QIDS-SR was used to evaluate baseline depressive symptom severity in a timeframe of 30 days around the diagnostic work-up, as part of routinely collected outcome measurements.

#### Dutch measure for quantification of treatment resistance in depression (DM-TRD)

The DM-TRD is an 11-item instrument combining information about the severity and duration of the current depressive episode, the level of functioning, the presence of any comorbid anxiety and personality disorders and psychosocial stressors with information about failed treatments including pharmacology and psychotherapy for the current episode [43]. A higher DM-TRD total score (range 2–27) is associated with worse treatment outcome [39, 43]. We used the total score of the DM-TRD and scores on its items to examine risk factors for dropout.

#### Standardized assessment of personality—abbreviated scale (SAPAS)

The presence of a comorbid personality disorder in the DM-TRD was based on the Standardized Assessment of Personality—Abbreviated Scale (SAPAS) [44] in subjects that were lacking a formal DSM-IV personality pathology diagnosis. The SAPAS is a clinician rated eight-item questionnaire that can be used to screen for the presence of personality disorders in depressive patients [45].

### Definition of dropout

Subjects were considered to be *dropout* when they did not show up for a follow-up session after 1–3 therapy sessions within six months after the start of the treatment. Because dropout mainly occurs at the beginning of the treatment [6, 7] and because it is conceivable that the patient has recovered sufficiently after 4 therapy sessions [46–48] we have opted for this approach. Therapy sessions at least consisted of a first face-to-face appointment in the context of pharmacotherapy (including supportive treatment elements as usual) or psychotherapy after the diagnostic work-up. In case of psychotherapy, follow-up sessions also had to be face-to-face contacts. In the case of pharmacotherapy, this could also be a follow-up consultation by telephone, as usual in such outpatient settings.

Dropout subjects that had a consecutive QIDS-SR measurement that showed remission (QIDS-SR < 6) were deemed to have stopped as *justified quitter* and were thereby not considered as dropouts in our analysis. Otherwise, regardless of the presence of a follow-up QIDS-SR measurement, the number of attended therapy sessions was leading in our dropout definition.

### Statistical analyses

The clinical and demographic characteristics of subjects and differences between subjects with and without baseline QIDS-SR-scores were assessed using descriptive statistics, Chi-square- and *t* tests as appropriate. Considering the large sample size, we calculated Cramér’s V in case of significance to interpret the clinical relevance of the effect.

To predict dropouts, we initially performed a logistic regression to investigate the relationship between dropout and total DM-TRD score. To further investigate the predictability of dropout, we entered gender, age, marital state, social economic state (SES) combined with the independent DM-TRD items in our model to perform penalized binary logistic regression in the form of the elastic net [49], using the R-package glmnet [50]. For an introduction to regularization methods in linear models, we refer to James et al. [51].

In brief, the elastic net can be seen as a hybrid approach blending ridge regression, which can handle correlated features but does not perform feature selection, and the lasso, which can perform feature selection but does not handle highly correlated features very well [52]. In ridge regression, weak independent variables will have their regression coefficients shrunken towards zero [53]. Lasso involves shrinking regression coefficients completely to zero eliminating them from the regression model [54]. For both methods, the proper amount of shrinkage is chosen using cross-validation, which can be regarded as a way to balance the bias–variance trade-off: a certain amount of bias is introduced into the regression coefficients (by shrinking), reducing the variance of the predicted values in such a way that the estimated classification error rate is minimized [51]. In elastic net, a trade-off between ridge and lasso regularization is made by weighing the penalties involved in both methods.

In our analysis, the choice of the weight parameter was made by means of tenfold cross-validation [55] with weights chosen over the complete unit interval between full ridge regression and full lasso, adhering to the one-standard-error-rule [55] to get to the method with the highest sparsity that lies within one standard error of the cross-validated prediction error from the optimal solution. Stability of the weight parameter was assessed by performing the tenfold cross-validation one hundred times. Because regression parameters tend to be biased towards zero, in a final step a regular logistic regression was performed, only containing features that remained in the elastic net procedure [52, 56].

Classification efficacy was assessed using the receiver operator characteristic (ROC) curve. Using every value of the predicted probability of becoming a dropout under the final model as a cut-off point, the true and false positive rates were assessed for that cut-off value. For a logistic regression model in general, the classification performance is assessed as a function of the area under ROC curve (AUC), where 0.70 ≤ AUC < 0.80, 0.80 ≤ AUC < 0.90 and ROC ≥ 0.90 imply acceptable, excellent or outstanding discrimination, respectively [57].

## Results

### Participants

**Fig. 1:**
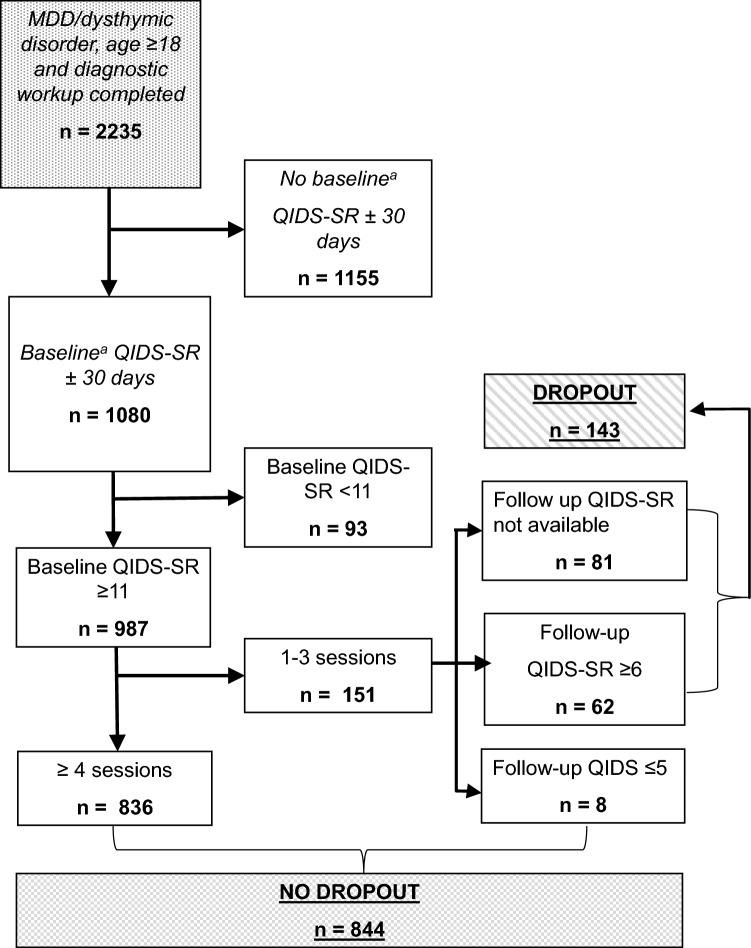
Study population flowchart. ^a^There were no clinically relevant differences in baseline characteristics between subjects with and without baseline QIDS-SR scores. *MDD* major depressive disorder, *QIDS-SR* quick inventory of depressive symptomatology—self-rating

According to the EPR, 2235 subjects met our inclusion criteria and had complete diagnostic work-up information concerning DM-TRD. Of this sample, 1155 subjects were lacking a QIDS-SR measurement at baseline. There were no differences between subjects with (*n* = 1080) or without (*n* = 1155) a baseline QIDS-SR score in age, SES and total DM-TRD-scores according to *t* tests (*t* ≤ 1.64, *p* ≥ 0.10). There was also no significant difference in these groups for gender between the groups with and without a baseline QIDS-SR scores (*χ*^2^(1) = 0.398, *p* = 0*.*53). There was a significant difference in the distribution of various marital states (committed relationship or not, unknown) between subjects with and without baseline QIDS-SR score (*χ*^2^(2) = 7.5, *p* = 0.023). However, according to the Cramer’s V (0.058), the clinical relevance of this significance was, due to the large sample size, very small. Therefore, the sample was assumed to be representative and the 1155 subjects without a QIDS-SR measurement at baseline were not included in the primary analyses. Of the 987 subjects who met the inclusion criterion of having a baseline QIDS-SR score of ≥ 11, 143 subjects (14.5%) met our criteria for dropout (Fig. 1). Demographic and clinical characteristics at baseline of the total sample and of the analyzed sample are described in Table 1.

**Table 1:** Clinical and demographical characteristics of the sample

	Total sample (*n* = 2235)	Analyzed sample (*n* = 987)	
		Dropout (*n* = 143)	No dropout (844)
	*n* (%)	*n* (%)	*n* (%)
Demographics			
Age, years (mean ± SD)	40.0 ± 12.5	37.7 ± 13	40.0 ± 12.4
Female gender	1488 (66.6)	94 (65.7)	555 (65.8)
Committed relationship	677 (30.3)	38 (26.6)	251 (29.7)
SES (mean ± SD)	− 0.5 ± 1.6^a^	− 0.8 ± 1.6^b^	− 0.5 ± 1.5^c^
Clinical characteristics			
QIDS-SR baseline mean (mean ± SD)^d^	16.9 ± 4.49	17.3 ± 3.5	18.0 ± 3.5
Total DM-TRD-score (mean ± SD)	9.26 ± 1.99	8.6 ± 1.8	9.4 ± 1.8
Current episode > 24 months	707 (31.6)	32 (22.4)	268 (31.8)
Severe depression ± psychotic symptoms	540 (24.2)	29 (20.3)	230 (27.3)
Functional impairment moderate-severe	1945 (87)	125 (87.4)	756 (89.6)
Comorbid anxiety	1358 (60.7)	76 (53.2)	519 (61.5)
Comorbid personality pathology (SAPAS or DSM-IV)	1444 (64.6)	78 (54.5)	569 (67.5)
Actual psychosocial stressors	1780 (79.6)	110 (76.9)	689 (81.6)
Treatment failures			
Antidepressant use (≥ 1)	939 (41.9)	46 (32.2)	374 (44.3)
Augmentation/combination	282 (12.6)	14 (9.8)	105 (12.4)
Psychotherapy	937 (41.9)	50 (35)	368 (43.6)
ECT	15 (0.7)	0 (0)	4 (0.5)
Intensified treatment			
Dayclinical	16 (0.7)	2 (1.4)	5 (0.6)
Inpatient	54 (2.4)	1 (0.7)	13 (1.5)

*DM-TRD* Dutch measure for quantification of treatment resistance in depression, *DSM-IV* diagnostic and statistical manual of mental disorders 4th edition, *ECT* electroconvulsive therapy, *SES* Socioeconomic status, *QIDS-SR* quick inventory of depressive symptomatology—self-rating, *SAPAS* standardized assessment of personality—abbreviated scale

^a^*n* = 2231 subjects

^b^*n* = 142 subjects

^c^*n* = 841 subjects

^d^*n* = 1080 subjects

### Total DM-TRD-score as predictor of dropout

A logistic regression analysis showed that a higher total DM-TRD-score was associated with lower odds for dropout (OR = 0.78, 95% CI = [0.70–0.86], *p* < 0.001). The classification performance according to the AUC was 0.53.

### Prediction of dropout by separate DM-TRD items and demographic parameters

In the prediction of dropout with the separate DM-TRD items, age, gender, marital status and SES, the optimal weight parameter was found at the end of the elastic net spectrum (*α* = 1.0), thus leading to the lasso penalty. Marital state, gender, the presence of psychosocial stressors and functional impairment were removed from the model. The predictors that remained in the lasso procedure were age, SES, duration and severity of the depression, the presence of comorbid anxiety and comorbid personality pathology and previously failed treatments (psychotherapy, anti-depressant use, inpatient and day hospital treatments). Parameter estimates with odds ratios and confidence intervals for the logistic regression containing these predictors can be found in Table 2. Higher SES, more severe depression, comorbid personality pathology according to the SAPAS and a comorbid DSM-IV anxiety disorder were significantly associated with less dropout.

**Table 2:** Estimates from logistic regression with features maintained in elastic net procedure

	Estimate	SE	*z* value	*p* value	OR	95% CI
Intercept	0.58	0.63	0.93	0.35	1.79	0.52–6.15
Age	− 0.02	0.01	−1.95	0.05	0.98	0.97–1.00
SES	− 0.20	0.07	−2.98	< 0.001*	0.82	0.72–0.93
Duration^a^						
Subacute	− 0.32	0.28	−1.14	0.25	0.73	0.42–1.26
Chronic	− 0.47	0.26	−1.82	0.07	0.63	0.38–1.04
Severity^b^						
Mild	− 0.85	0.58	− 1.48	0.14	0.43	0.14–1.32
Moderate	− 0.95	0.49	− 1.92	0.06	0.39	0.15–1.02
Severe	− 1.30	0.54	− 2.41	0.02*	0.27	0.09–0.78
Severe with PS	− 2.01	1.16	− 1.73	0.08	0.13	0.01–1.30
Comorbid anxiety						
DSM diagnosis −	− 0.10	0.23	− 0.42	0.68	0.91	0.58–1.43
DSM diagnosis +	− 0.92	0.35	− 2.64	0.01*	0.40	0.20–0.79
Comorbid PP						
SAPAS ≥ 3	− 0.44	0.22	− 1.98	0.05*	0.65	0.42–0.99
DSM diagnosis +	− 0.66	0.78	− 0.85	0.39	0.51	0.11–2.38
Treatment failures						
Antidepressants	− 0.33	0.24	− 1.36	0.17	0.72	0.45–1.15
Psychotherapy	− 0.41	0.23	− 1.80	0.07	0.66	0.42–1.04
Day hospital	1.65	0.89	1.84	0.07	5.19	0.90–29.96
Inpatient	0.51	1.10	0.46	0.64	1.66	0.19–14.40

*DSM* diagnostic and statistical manual of mental disorders, *PS* psychotic symptoms, *PP* personality pathology, *SAPAS* standardized assessment of personality—abbreviated scale

^a^Reference category = “acute (< 12 months)”

^b^Reference category = “subclinical”

*p < 0.05

**Fig. 2:**
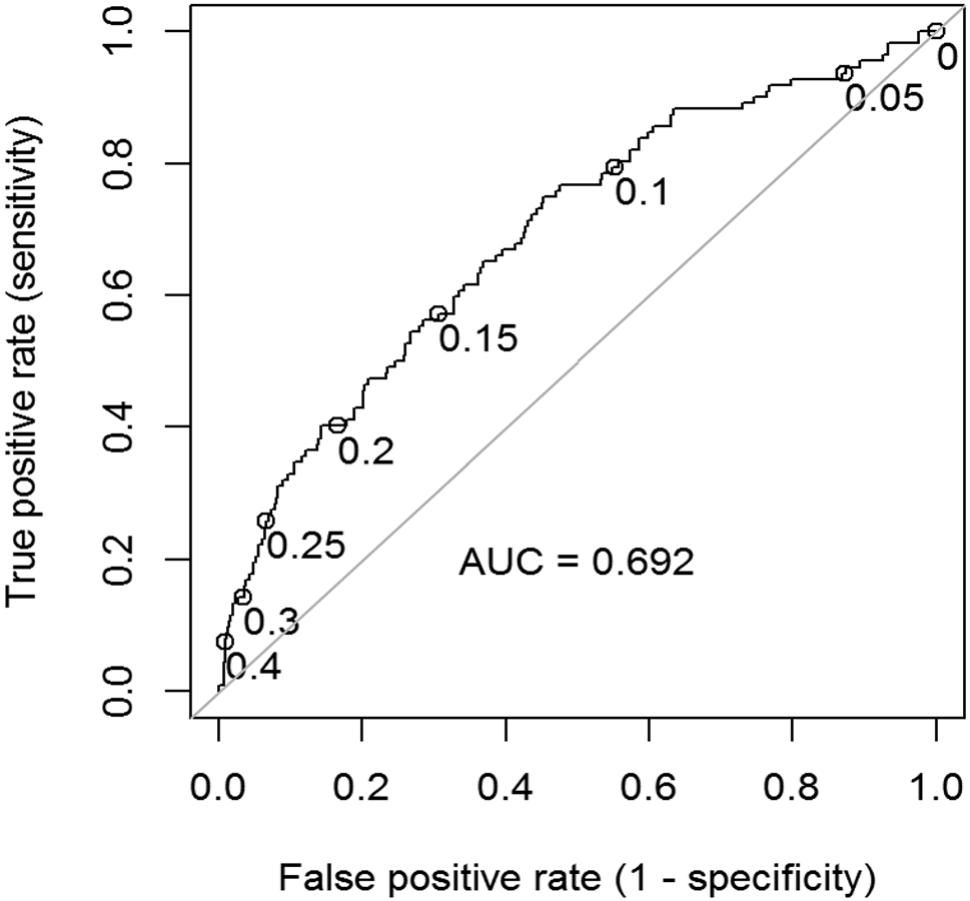
Receiver operating characteristic curve for logistic regression model with features maintained in elastic net procedure. Some cut-off points are depicted on the curve. *AUC* area under the curve

For both groups, the mean predicted probability of dropout according to our model was low: 12.6% for the non-dropout group and 19.2% for the dropout group. The ROC curve for this model can be found in Fig. 2. There is no evident cut-off value for the predicted probability where both sensitivity and specificity are high. For instance, with the cut-off value of 0.1, the sensitivity of the model is 79.5%, but the specificity is only 45.0%, meaning a false positive rate of 55.0%. Furthermore, the AUC value of 0.692 indicates that the ability of our final logistic regression model to distinguish between dropout and non-dropout is below the acceptable threshold.

## Discussion

In this study, we examined prevalence and risk factors for dropout in routine care in a large sample of outpatients with depression receiving routine outpatient depression treatment. We found a dropout rate of 14.5%. Higher DM-TRD total scores were predictive for lower odds for dropout, although it was not possible to determine a cut-off score to predict accurately whether or not an individual would dropout. When analyzing separate risk factors for dropout, higher SES, higher severity of the depression and the presence of comorbid personality pathology and a comorbid anxiety disorder at baseline were associated with a decreased risk of dropout in our sample.

The prevalence of dropout in our sample was lower compared to the overall prevalence rates of 20–25% as reported in recent meta-analyses [6, 9, 36]. However, it should be noted that these meta-analyses identified very wide ranges in observed dropout between 0 and 50–74%. As such, the dropout rate in our study appears low but in line with results from earlier work in RCTs and other naturalistic samples [22, 27, 58]. It is unknown whether our setting and therapeutic approaches play a role in our dropout rate. Our participating centers are part of a large secondary care mental health organization, a type of setting associated with lower dropout rates than academic or university affiliated centers [9]. More detailed comparison with other studies is impossible given the differences between definitions of dropout that are being used in the literature. We chose to define participants as dropouts when they had attended only 3 or less treatment sessions. Although some of the patients with MDD may benefit from psychotherapy within a few sessions [46], the majority of patients needs at least four sessions to achieve an adequate outcome [47, 48]. Additionally, previous work showed that, in the Netherlands, an acute phase pharmacological treatment for depression typically entails five or six visits to a psychiatrist [59]. It can be expected that raising the required ‘at least 4’ sessions or defining dropout as 'not attending any session after diagnostic assessment' would have increased dropout rates erroneously.

The DM-TRD is an easy-to-use clinician rated instrument combining clinical characteristics with information about previous treatments for the current depressive episode. We found that higher total scores on the DM-TRD were associated with less dropout. This was somewhat unexpected because the DM-TRD is designed to assess the risk for treatment resistance, and consists of items that score previous failed treatments for the index-episode, as well as clinical risk factors for treatment resistance including severity and duration of the episode and comorbid psychopathology. We hypothesized that higher scores on a composite of these items would be associated with less motivation for treatment and more pessimism about treatment success and as such expected an association with higher dropout rates. Our results, however, point in the opposite direction; one could speculate that higher levels of treatment resistance at baseline increase motivation and presumably the need for treatment in an otherwise unfavorable course of illness.

In line with previous findings [14, 15] higher SES was associated with less dropout in our sample. Inconsistencies about the association of dropout with demographical features age, gender and marital state that emerge from the literature (e.g., [6, 9, 13, 17]) may explain why these features were not predictive for dropout in our sample. In addition, this result can be attributed to the fact that the other variables contributed more to the dropout variance than these demographic characteristics.

Contrary to our hypothesis, the clinical variables severity at baseline and duration of the depressive episode were negatively or not associated with dropout. Comparison of this finding with meta-analyses that examined treatment dropout is difficult because baseline severity and episode duration were not assessed [6, 9, 36, 60]. As previously described, the association between severity and dropout is not unambiguous (e.g., [13, 23, 27]). The finding that more severe patients were less likely to drop out is in line with several studies [8, 22, 23]. A possible explanation for this finding is that this is a result of the way care is delivered in our setting. As proposed by Fernández et al. [8], a mismatch of treatment intensity with depression severity appears to be a causative factor for dropout. This is supported by the fact that patients with more severe depression are more likely to drop out from a self-guided online treatment intervention [16]. Our institution offers treatments from a multidisciplinary team consisting of psychiatrists, psychologists, nurses and other mental health professionals. It is therefore conceivable that in our setting a good match has been made with the needs of the patient, resulting in less dropout in the more severely depressed patients. In line with Machado et al., there was no association between more chronicity and the occurrence of dropout in our sample [21].

Previous meta-analyses showed higher dropout rates in patients with personality disorders [6, 9], while other studies showed that comorbid personality pathology had no negative effect on dropout rates in the treatment of depression [13, 21]. In our sample, the presence of comorbid personality pathology according to the SAPAS, but not the presence of a personality disorder as established by a structured clinical interview as the SCID-II, was associated with a lower risk for dropout. A probable explanation for this is that the patient's responses to the items of the questionnaire were negatively influenced by the severity of the depression due to its negative effects on self-perception (the state-effect) and memory processes [61, 62]. The reported personality pathology should thereby perhaps be seen as a proxy for the severity of the depression, which in turn was associated with lower dropout rates in our sample. The fact that there was no association present for an actual personality disorder according to a clinical interview supports this conjecture. Another relevant aspect is that the type of personality pathology appears to be relevant for the occurrence of dropout. For example, the presence of compulsive personality traits can have a protective effect on the occurrence of dropout [19, 21]. Since cluster C is the most common cluster of comorbid personality pathology in MDD [63], this could also explain the association of comorbid personality pathology with less dropout in our sample. Unfortunately, due to the way personality pathology was assessed in our sample, it was not possible to accurately distinguish between the various clusters.

We found that the presence of a comorbid anxiety disorder but not the presence of anxiety symptoms was predictive for lower dropout rates. This finding is not in line with previous studies reporting higher dropout rates in depression treatment in subjects with comorbid anxiety disorders [13, 30]. This inconsistency may be explained by the fact that combination treatments are mainly offered in our treatment setting. Combination therapy showed less dropout than pharmacotherapy alone in subjects with comorbid anxiety disorders [13]. In addition, some studies found that patients with MDD and comorbid anxiety disorders can experience a faster recovery from psychotherapy [29, 64]. Since patients who showed less symptom reduction during treatment for depression were more likely to drop out [1, 29], these factors may be related to the finding that a comorbid anxiety disorder is predictive of less dropout in our sample.

The divergence for both comorbidities may also be explained by the specialized secondary healthcare settings of PsyQ, where patients are referred for depression treatment specifically. When personality pathology or an anxiety disorder is strongly present, specialized treatment is offered aimed at these specific disorders. Moreover, therapists within these specialized treatment settings are used to offering MDD treatment to patients with comorbidities. By adapting the way the MDD treatment is offered by these therapists to the needs of the patient, the risk of dropout can be reduced, as suggested by Swift and Greenberg [65].

Several limitations need to be mentioned. First, although the naturalistic setting is an overall strength, this limited the possibility to obtain more information concerning the specific types of treatments, adherence and treatment integrity. We also do not have information on the content of the planned treatment and the reasons for dropout. It cannot be ruled out for certain that in some cases the treatment termination was a joint decision between patient and therapist. However, we do not consider this likely, as the population had severe complaints (QIDS-SR ≥ 11) that are not expected to remit within 1–3 sessions. In addition, subjects who had 1–3 therapy sessions, but whose follow-up measurement on the QIDS-SR showed remission, were defined as “justified quitters” and were therefore not considered to be dropouts. Unfortunately, we also do not have information on the course of the dropouts after the 6-month time frame that our study focused on. It is conceivable that some of these dropouts eventually returned with a renewed request for help in the same or another treatment setting. However, we lack this information and we think this could be a relevant question for future studies on this topic. Second and related, this lack of information on the content of the treatment and the reasons for dropout prevents a more detailed analysis of an association between treatment type and dropout, and their potential interaction with sociodemographic variables; this would be relevant given the difference in dropout between psychotherapy and pharmacotherapy [10, 60]. Third, it may be that an unmeasured variable (e.g., more previous episodes [28]) in more severe and chronic cases drives the association with less dropout. More recurrent episodes may make subjects realize treatment is necessary from past experience. Likewise, information on other relevant patient, treatment and therapist-related variables was absent [9, 32, 33, 35]. Previous work has shown that, apart from demographics and clinical characteristics, a variety of factors may contribute to dropout, although results are again not in complete accordance. These factors include patient and treatment-related variables such as patient’s treatment preference, (pre-) treatment expectancy, treatment setting, treatment format, manualized versus non-manualized treatment, treatment duration and therapeutic alliance, but also therapist-related information such as gender, age, patients’ treatment preference, experience level, and the therapist’s ability to repair alliance ruptures [9, 32, 33, 35, 66, 67]. Unfortunately, this information was not available. Fourth, the presence of personality pathology (in the absence of a SCID-II) was determined in our sample using a concise questionnaire, the SAPAS. Diagnostics based on a questionnaire can lead to over-reporting of personality pathology compared to diagnostics based on a clinical interview [63]. It is thereby conceivable that the way personality pathology was assessed in our sample had led to an overestimation of this comorbidity. In addition, it was not possible to distinguish between the specific types of comorbid disorders, both within the personality pathology and within the anxiety disorders. Fifth, although our sample was large, a substantial number of subjects could not be analyzed due to lacking baseline QIDS-SR scores according to our definition of 30 days around diagnostic work-up. In addition, it is conceivable that the lack of a baseline measurement is already indicative of the occurrence of dropout. On the other hand, there were no clinically relevant differences at baseline parameters between the analyzed sample and the subjects that were missing QIDS-SR scores. Fifth, it is conceivable that, due to missing follow-up QIDS-SR measurements, a part of our subjects was falsely qualified as “dropout”. However, given the natural course of MDD [68] and given the baseline severity of our subjects, it is not very likely that a substantial part of these patients would have recovered without adequate treatment.

## Conclusions

In this observational study dropout from depression treatment was relatively low in comparison to previous studies and meta-analyses. We found that the DM-TRD was useful to assess dropout risk: higher total scores on this instrument were associated with a lower dropout rate. Several clinical variables from the DM-TRD, i.e., higher socio-economic state, higher severity of the depression and the presence of comorbid personality pathology and comorbid anxiety disorders, were significantly associated with a lower dropout risk. The DM-TRD may, therefore, be helpful to clinicians to assess risk factors for dropout before treatment commences.

## Footnotes

1 Zoetermeer, Zaandam, Rotterdam Kralingen, Rotterdam South, Beverwijk, Spijkenisse and The Hague.
